# Anemia and associated factors among internally displaced children at Debark refugee camp, North Gondar, Northwest Ethiopia

**DOI:** 10.1371/journal.pone.0285627

**Published:** 2023-05-10

**Authors:** Bisrat Birke Teketelew, Biruk Bayleyegn, Dereje Mengesha Berta, Bamlaku Enawgaw, Berhanu Woldu

**Affiliations:** Department of Hematology and Immunohematology, School of Biomedical and Laboratory Sciences, College of Medicine and Health Sciences, University of Gondar, Gondar, Ethiopia; Wollega University, ETHIOPIA

## Abstract

**Background:**

Children in refugee camps, due to their living conditions, are the most vulnerable groups to suffer from anemia. Nutritional deficiencies, especially iron deficiency is the most common causes of anemia. However, there is limited information on the prevalence and associated factors of anemia in Ethiopia. Hence, this study aimed to assess the prevalence and associated factors of anemia among internally displaced children at Debark refugee camp, Northwest Ethiopia.

**Methods:**

A cross-sectional study was conducted on 354 internally displaced children, at Debark refugee camp from March to May 2022. A systematic sampling technique was employed. The socio demographic characteristics were collected by using structured questionnaire via face-to-face interview with the caregivers. The hemoglobin level was determined using HemoCue301+. Form anemic children, venous blood sample was collected for iron profile analysis. Parasitological and anthropometric measurements were also done. The data were entered using Epi-data version 4.6.0.6 and exported to STATA version 14 for analysis. Bi-variable and multivariable binary logistic regression analysis were done. Both crude odds ratio and adjusted odds ratio with the corresponding 95% confidence interval were calculated to measure the strength of association. P-Value < 0.05 was considered as statistically significant association.

**Results:**

From the total of 354 children included in this study, more than half (54.8%) of them were male. The median age of children was 7 years with interquartile range of (4–10) years. The total prevalence of anemia in this study was 33.62% (95% CI:28.7, 38.7). Moderate type anemia was predominant in this study. From anemic children 30 (25.2%) had iron deficiency anemia. In this study, low dietary diversity (AOR = 4.9; 95% CI: 2.0, 11.7), duration in the camp more than six months (AOR = 4.2; 95% CI:1.9, 9.4), presence of diarrhea (AOR = 2.7; 95% CI:1.3, 5.7), fever (AOR = 3.4; 95% CI:1.6, 7.1), and wasting (AOR = 3.6; 95% CI:1.3, 10.3) were significantly associated with the prevalence of anemia.

**Conclusion:**

Anemia was moderate public health problem in the current study. Focused policies and strategies towards to internally displaced children should be designed to reduce anemia, by preventing the significant risk factors associated with anemia.

## Introduction

Anemia is defined as a decrease in the red blood cell number, hemoglobin content of red blood cells (RBCs), and hematocrit value below the reference interval for healthy individuals of similar age, sex, and race [1]. The World Health Organization (WHO) defines anemia in a population as a mild, moderate, or severe public health problem if the prevalence is 5–20%, 20–40%, and >40%, respectively [2].

On a biological level, anemia is produced by an imbalance in erythrocyte loss and formation [3]. This is due to acute or chronic blood loss, reduced or impaired RBC production, and increased the destruction of RBCs [4]. It is the result of a wide variety of causes that often coexist together. The most significant contributor cause is dietary iron deficiency (ID) especially in fast growing children [5]. The pathophysiological mechanism for the development of anemia in children mainly depends on the disequilibrium between rapid growth iron intake [6]. The abundant iron stores present at birth help to provide for the synthesis of hemoglobin and enzyme iron during the first four months of life. However, after about four months of age, a gradual shift occurs from an abundance of iron to the marginal iron. The transition from feast to famine with respect to iron is primarily due to the large amount of iron required to maintain a near constant mean hemoglobin concentration of 12.5 g/dl within a rapidly expanding blood volume [7].

Anemia is an important cause of health loss throughout the world, approximately 1.8 billion people are affected across the world [8] and half of them are suffer from IDA [2]. By the end of 2019 anemia affects 39.9% of the global children [9]. Displaced persons particularly children have a unique risk for developing anemia with estimated global prevalence of 43% [10]. The highest experienced prevalence of anemia is in refugee camps of west Africa (Nigeria and Cameroon is 54% and 84%, respectively), central Africa and sub-Saharan Africa, especially in Kenya and Ethiopia (52.4% Somalia region, Ethiopia [11–13].

Children in refugee camps and emergency areas are extremely vulnerable to anemia and other micronutrient deficiencies. This is due to inadequate food rations, limited micronutrient composition, limited access to health services, unhealthy environment, poor feeding and caring practices, overcrowding, uneven access to clean water, increased exposure to infectious diseases and lack of other specialized services needed by displaced individuals [14, 15]. Relying on food aid to meet nutritional needs may also raise the risk of micronutrient deficiencies and anemia, particularly among people living in long-term camp settings [15].

The common outbreaks of communicable diseases in refugee camps among vulnerable groups are linked to high levels of malnutrition which is a major contributor to childhood anemia [16]. High incidences of severe malnutrition have been reported in the camps. Such levels of malnutrition raise serious concern among displaced children because of the recognized synergism between poor nutrition and disease occurrence [12]. As ID is one of the common type of malnutrition, such children can suffer from IDA, which is the major health consequence of intellectual disabilities [2]

Iron deficiency anemia has serious short- and long-term repercussions for children’s health, including developmental delays, impaired cognitive development (lower learning and school performance), loss of recent and past memory concentration, loss of interest to the environment, diminished behavioral problems, fatigue, difficulty focusing, lethargy, and infection vulnerability [2]. Different studies reported that IDA in children has been strongly correlated with psychiatric disorders and mental retardation [17, 18]. Furthermore, IDA in children is linked to a reduced ability to fight infections due to a lowered immune system [14].

Laboratory tests, in conjunction with physical examination, are critical in diagnosing, determining, and monitoring the appropriate treatment of anemia. Typically, the evaluation of the cause of anemia includes, evaluations red cell parameters, peripheral morphology examination, reticulocyte count, immature reticulocyte fraction and serum iron indices test [19, 20]. Based on this diagnosis, the management is continued by considering the type of anemia, age of the patient, symptoms and general health [8].

Currently, an estimated 5.6 million people were displaced in Ethiopia due to armed conflict and natural disasters [21]. These reports account for approximately 5.6% of UNHCR’s global internal displacement report [22]. Although the burden of anemia is high among refugee children due to their living conditions, its prevalence and associated factors are not yet sufficiently identified and documented in Ethiopia in general and in the study area in particular. Iron deficiency anemia is common in fast-growing and infectious-risk children, but limited studies were shown the prevalence of IDA among children in displaced settings. Lack of current reports on the prevalence of anemia and IDA among children at refugee camps in Ethiopia, may limit the rate at which decisions are made for intervention measures by concerned bodies such as national government, international organizations and health care providers. Therefore, the main aim of this study was to assess the prevalence of anemia and IDA among IDP children at Debark refugee camp.

## Methods and materials

### Study area

This study was conducted at Debark refugee camps which is located in Debark town, North Gondar, Northwest Ethiopia. Debark town is found in North Gondar Zone of Amhara National Regional State. It is located 837 km far from Addis Ababa, the capital city of Ethiopia, 279 km from Bahir Dar city and 99 km from Gondar town. Debark has a latitude and longitude of 13°08′N 37°53′E and an elevation of 2,850 meters above sea level [23]. Currently the camp is known for hosting more than 2800 internally displaced persons from different districts of Amhara regional state after emergence of conflict between Tigray forces and Ethiopia’s central government. About 40% of the displaced person in the camp are children [24].

### Study design and period

A cross-sectional study design was employed from March to May 2022 to assess the prevalence and associated factor of anemia among internally displaced children.

### Population

All children aged between 6 months and 15-year live in Debark refugee camp were used as source population. And children, who fulfill the inclusion criteria were considered as study population. Children aged between 6 months and 15 years and live in the camp with a minimum of three months were included in the study. However, children aged between 6 months and 15 year who had previously diagnosed anemia and on medication, critically ill children, having history of recent transfusion, children on iron supplementation were excluded from the study.

### Study variables

Anemia was considered as dependent variables. On the other hand, Sociodemographic variables like child age, sex, maternal/care giver’s educational status, marital status, house hold size, number of children per household, source of drinking water, camp duration. Clinical variables (intestinal parasite, malaria, diarrhea, vomiting, fever), feeding characteristics (meat consumption, egg consumption, fruit and vegetable consumption, milk intake, intake of fortified food), anthropometric variables like stunting, wasting and dietary diversity, ration duration were also considered as independent variables.

### Sample size determination and sampling technique

The sample size was calculated using single population proportion formula (n = [(zα/2)^2^ × (1-p)]/d^2^) with a 95% level of confidence and 5% margin of error. Since we did not get any current information on the prevalence of anemia among internally displaced children in Ethiopia, a 50% proportion was considered.


$$\text{n}=\frac{{\left(Z\frac{a}{2}\right)}^{2}\;x\;P\left(1-P\right)}{d2}=\frac{{\left(1.96\right)}^{2}\;x\;0.5\left(1-0.5\right)}{{\left(0.05\right)}^{2}}=384$$

Since, the population at Debark refugee camp (2,800) is below 10,000 the sample size was corrected by using correction formula and given to 338. After added 10% nonresponse rate the final sample size, 372 was considered.

Correction formula:

$${\text{n}}_{\text{f}}={\text{n}}_{\text{o}}/1+\left({\text{n}}_{\text{o}}-1/\text{N}\right)=384/1+\left(384-1/2800\right)=338$$

Where; n_f_ = final sample size, n_o_ = sample size before correction, N = total number of populations

After added non response rate 338+ (338x0.1) = 372. Debark refugee camp has 1545 households. From these 1,120 households were identified as presence of children. Once the study population is identified then systematic random sampling was employed to select the required sample size. The first study participant was selected by lottery method. Sample frame was prepared from the list of data base from Debark town food and security bureau. The K value was derived as;

K = Number of households with children/n = 1,120/372 = 3. Children were selected systematically in every three intervals up to the required sample was obtained.

### Operational definition

#### Anemia

Anemia is defined according to the WHO as a hemoglobin concentration of less than 11 g/dL for children aged 6 to 59 months, less than 11.5 g/dL for children aged 5 to 11years, and less than 12 g/dL for children aged 12 to 14 years after altitude adjustment of hemoglobin to sea level. Since Debark has an altitude of 2,850 meter a hemoglobin value of 1.9 g/dL was subtracted from the measured value to adjust the hemoglobin value (Adjusted hemoglobin = Hemoglobin measured value– 1.9g/dL [25]. Moreover, anemia severity was classified as mild: when: Hgb 10.0–10.9 g/dl for children aged 6–59 months, 11.0–11.4 g/dl for children aged 5–11 years and 11.0–11.9 g/dl for children aged 12–14 years. Moderate, when Hgb 7.0–9.9 g/dl for children aged 6–59 months and 8–10.9 g/dl for children aged 5–14 years. And Severe when Hgb <7.0 g/dl for children aged 6–59 months and <8.0 g/dl for those aged 5–14 years [25].

### Data collection and laboratory method

Socio-demographic characteristics of children (age, gender, number of children per house hold) and maternal/care giver related socio-demographic characteristic (age, marital status, educational status) as well as socio demographic characteristics of HH (household size, source of drinking water), clinical characteristics of children (diarrhea, fever, vomiting, malaria infection, intestinal parasite) and dietary information like consumption of meat, egg, fruit and vegetable, milk was assessed. The individual dietary diversity score also was assessed. All the information’s were collected using a structured questionnaire via a face-to-face interview technique from children’s mother or caregiver. The socio demographic questionary was developed by assessing different literatures modified into local terms.

#### Food dietary diversity assessment

Food dietary diversity was assessed with a questionnaire consisting of twelve groups of food items. The food items were grouped into cereals, legumes, fruit, vegetables, iron rich organ meat, flesh meat, egg, fish and sea foods, milk and milk products, oil and fats, sweets, and other foods, such as spices, condiments. Some food items in the dietary diversity questionnaire were combined into a single food group to create nine individual dietary diversity groups. Then dietary diversity scores (DDS) were calculated from these food groups and categorized as high (DDS ≥ 6), medium (DDS = 4–5), and low (DDS ≤ 3 as per food and agriculture organization (FAO’s) guideline [26].

#### Anthropometric measurements

The data indicators used for anthropometry in this study were age, height, weight and mid upper arm circumference (MUAC). The summary indices of nutritional status, Weight for height (wasting) and Height for age (stunting) were calculated as per recommended by WHO [27]. The weight of children was measured while wearing light-weight cloth with a portable weight scale to the nearest 0.1 kg. The weighing scale was calibrated using the standard calibration weight of 2 kg iron bars, whereas the precision was checked by measuring children’s weight twice and took the average. Child height was measured using a locally manufactured wooden stadiometer with a sliding head bar to the nearest 0.1 cm in Frankfort position (head, shoulder, buttocks, knee, and heals were touch the vertical board). Weight and height of children less than two years old were measured in recumbent position. On the other hand, weight and height of the children who were more than two years old were measured in standing position.

Then *Z*-scores of nutritional indices, such as height-for-age (HAZ) was calculated using WHO Anthro version 3.2 (for children aged ≤5 years) and Anthro-plus version 1.0.4 (for children aged >5 years) soft wares. Finally, children were classified as stunted when the HAZ scores was less than -2 standard deviations (SD) [27], and wasted when the MUAC value was <12.5cm for children less than 5 years, <14.5cm for children 5–9 years and <18.5 for children 10-14years. The cut of value for MUAC is based on Nutrition Assessment, Counseling, and Support (NACS) user guide [28]. Those all-available data were collected by trained clinical nurses working in the camp.

After getting informed consent and assent, blood sample was collected following standard operating procedures (SOPs) by an experienced laboratory technologist. A ring finger was selected and cleaned with 70% alcohol. Then finger prick was done to take capillary blood for Hb determination. Children with low Hb level (i.e., anemic) were selected for vein puncture blood sample collection. Three milliliter of blood sample were collected from median cubital vein, and heel with a sterile disposable syringe and butter fly technique after cleaning the site with 70% alcohol. Then it was delivered into serum separator tube for analysis of iron profiles.

#### Hemoglobin determination

The Hb concentration of each participant was measured and determined immediately on-site by taking a finger-prick blood sample using a portable hemoglobinometer instrument (HemoCue 301+, Ängelholm, Sweden), which is recommended by WHO for the use of field surveys [29]. A blood sample of approximately 10 μL is drawn into the cavity of HemoCue301+ microcuvette by capillary action. Immediately the cuvette was placed to HemoCue301+ an absorbance of whole blood at a Hb/HbO2 isosbestic point was measured at two wavelengths (506 and 880 nm). The Hb level was then calculated and presented. Hb was adjusted for altitude as per WHO recommendation [25]. As Debark has an altitude of 2,850 meters above sea level a hemoglobin value of 1.9g/dl was subtracted from each Hb concentration measurements.

#### Serum iron profile test

All anemic children with HemoCue 301+ were considered for serum iron profile tests to determine IDA. For determination of serum iron profile, the blood samples in the test tubes were immediately wrapped in aluminum foil, continually shaded from light, and stored for 30 min at 4°C until centrifugation. Serum was centrifuged, kept in plastic screw-capped bottles labeled with the participant’s name, and kept at 20°C. Frozen serum samples were shipped using ice box to the University of Gondar comprehensive and Specialized Hospital clinical chemistry laboratory for the analysis of iron profile. After the samples were reached in clinical chemistry laboratory, SF level was analyzed spectrophotometrically using Beckman Coulter DxI 800 automated clinical chemistry analyzer. The SI level was analyzed using Beckman Coulter DxC 700 automated clinical chemistry analyzer. The absorbance of SI was measured at 540nm. Furthermore, C-reactive protein (CRP) latex agglutination test was performed to rule out the presence of inflammation or infection. Iron deficiency anemia was defined as based the final concentration of SI and SF as SI level < 8.95 μmol/L and < 7.16 μmol/L with age of ≥ two years and <2 years old, respectively, along with as serum ferritin (SF) level of <11μg/L.

#### Hemiparasite examination

Thick and thin BF were prepared from capillary blood of febrile children on the same slide. To prepare thick film, approximately 6 μL of capillary blood was placed to precleaned and labeled slide. Using the corner of a clean slide, spread was done in circular manner with approximate diameter of 1–2 cm. to prepare thin film, approximately 2–3 μL of capillary blood was placed and spread with another clean slide at an angle of 45°. After air drying, the thin film was fixed with methanol and both films were stained using 10% Giemsa stain for microscopic examination of malaria parasite. After staining, the presence or absence of hemiparasites were detected on thick film. To identify the types of species, thin blood film was examined for those parasites detected on thick film. Both films were examined under 100x objectives.

#### Intestinal parasites examination

Each study participants were instructed to bring a pea-sized stool (approximately one gram) sample with the help of their mother/care giver. A stool samples were collected with a clean, leakproof and coded container. Then, the stool samples were transported to debark health center laboratory. Fresh stool sample was used for wet mount preparation and examined by well experienced laboratory technologist for the presence of intestinal parasites. Samples were preserved by 10% formalin, if not examined within 1 hour of collection.

### Data quality control and quality assurance

The questionnaire was prepared in English and translated to Amharic then back to English to check consistency. Training was given for the data collectors about the objective, relevance of the study, confidentiality of information, and study participants’ rights before actual data collection and close supervision was made during the data collection period. To standardize the questionnaire, pretest was conducted on 5% (19 children) of the sample size outside of the study area among internal displaced children at keberomeda camp located near to Azezo, Gondar, Ethiopia, before the actual data collection was take place. Blood sample quality was ensured by collecting and processing according to the SOP. Samples was checked whether they are in the acceptable criteria like, free of hemolysis, sufficient volume, correct labeling and collection time. Safety procedure and `specimen handling procedures were strictly followed for all tests (Hb, iron profile, BF, and stool examination). Built-in self-test was done to cheek the quality and sensitivity of the HemoCue 301+ hemoglobinometer. The performance of Beckman Coulter chemistry analyzer was checked by running appropriate quality control materials a minimum of once a day by laboratory personnel. The quality of wet mount was checked for ability to read small print through it. All laboratory reagents were checked for their expiry date. Stanning reagents were checked for their quality by using known malaria positive slide.

### Data analysis and interpretation

The data was entered into epi- data version 4.6.0.6 and it was checked for its completeness and consistency before entering for analysis. Then, the data was exported to a STATA version 14.0 for analysis. Shapiro Wilk test was done to check the normality of the data. Descriptive analysis like frequencies, percentages, medians and interquartile range (IQR) were calculated using the software. Tables and bar graphs were used for presentation of the results. To measure the association between the dependent and independent variables bi-variable and multi-variable binary logistic regression were used. Variables with a *p* value less than 0.25 in the bi-variable analysis were fitted into the multi-variable binary logistic regression analysis to control the possible effect of confounding. Hosmer Lemeshow test was done to check model goodness of fit. Both crude odds ratio (COR) and adjusted odds ratio (AOR) with the corresponding 95% confidence interval (CI) were calculated to measure the strength of association. Finally, in the multivariable analysis, variables with a *p* value less than 0.05 were considered as statistically significant.

### Ethical consideration

This study was conducted after an ethical approval was obtained from research and ethics review committee of the school of biomedical and laboratory sciences, college of medicine and health sciences, University of Gondar with ethical clearance reference letter of SBMLS 191. Furthermore, support and an official letter was written from North Gondar zone health bureau to the study camp administrative for cooperation and to whom it may concern to permit the study which is intended on the prevalence and associated factor of anemia among refugee children in debark refugee camp, Northwest Ethiopia. The purpose of the study was explained to the study participants and care givers prior to consent. We inform about the right to interrupt the interview and to refuse blood collection. We told them that the blood drawn was only used to measure Hgb levels in front of the study participants and after the result was measured and recorded. Written informed assent was sought from children’s caregiver and informed consent was also sought from the mother for their volunteer. The assent was taken in front of the mother or care giver and they were signed on the consent after detailed explanation of the procedure and the purpose of the study. Participation in the study were purely on voluntarily basis and rejection was possible. To ensure confidentiality of data, study subjects were identified using codes, and only authorized persons were accessing the collected data. Any abnormal findings related to child health, like severe and moderate anemia, children infected with parasites were linked to the camp health care providers for special treatments.

## Results

### Socio-demographic characteristics of IDP children at Debark refugee camp

A total of 354 study participants with 95.2% response rate were involved in this study. Lack of interest, fear of pain, unavailability of the camp during the data collection period were the common reasons for non-response.

In this study the median (IQR) age of children was 7 (4–10) years. Nearly half of the study participants were within the age group of 5–11 years old 187 (52.82%), and 194 (54.8%) were males. The average family size was mean ± SD (4 ± 2) persons per household. Average children per HH was mean ± SD (3 ±1) and about 156 (44.07%) of the HHs had two children. Most of the study participants were stayed in the camp more than 6 months 188 (53.1%) and the median duration of children in the camp was 6 months with IQR (5–7) (Table 1).

**Table 1:** Socio demographic characteristics of children and their mother’s/care giver’s among IDP at Debark refugee camp, 2022.

Variables	Category	Frequency (N)	Percentage (%)
Sex of children	Male	194	54.8
	Female	160	45.2
Age of children	6-59months	107	30.23
	5-11years	187	52.82
	12-14years	60	16.95
Number of children per HHs	1	55	15.5
	2	156	44.1
	> = 3	143	40.4
Duration in the camp	3–5 months	166	46.9
	6 months and above	188	53.1
HHs size	< = 3	128	36.16
	4–5	186	52.54
	> = 6	40	11.30
Care giver educational status	Unable to read and write	162	45.8
	Primary education	149	42.1
	Secondary education and above*	43	12.2
Care giver’s marital status	Married	275	77.7
	Single*	79	22.3

HHs = household size, Secondary education and above* indicates including college and university

Single* = includes divorce, unmarried, widowed

#### Clinical characteristics, feeding and anthropometric measurements of the study participants

From study participants 123 (34.73%) and 142 (40.11%) had diarrhea and fever in the last two weeks of the data collection period, respectively. One hundred six (29.94%) and 2 (1.42%) of the children were infected with intestinal parasites and malaria parasites, respectively. In this study, 106 (29.9%) of refugee children had one or more of the following intestinal parasites (*A*.*lumbricoides*, *G*.*lambilia*, *E*.*histolotica*, and *hookworm*). From anemic children 66 (55.46%) of the study participants had pallor mostly on their conjunctivae 58 (87.8%). Rice and wheat were the most stapled food among IDP children. Majority of the study participants 150 (42.4%) had low dietary diversity score (Table 2).

**Table 2:** Clinical, feeding and anthropometric characteristics of IDP children at Debark refugee camp, 2022.

Variable	Category	Frequency (N)	Percentage (%)
Height-for-age status	Stunted	140	39.55
	Normal	214	60.45
MUAC	Wasted	43	12.15
	Normal	311	87.85
Presence of diarrhea	Yes	123	34.75
	No	231	65.25
Presence of vomiting	Yes	26	7.34
	No	328	92.66
Presence of fever	Yes	142	40.11
	No	212	59.89
Intestinal parasites	Present	106	29.94
	Absent	248	70.06
Malaria parasites	Present	2	1.41
	Absent	140	98.59
Meat consumption	Yes	70	19.8
	No	284	80.2
Fruit and vegetable consumption	Yes	28	7.9
	No	326	92.1
Egg consumption	Yes	85	24.0
	No	269	76.0
Milk intake	Yes	32	9.1
	No	322	90.9
Dietary diversity score	Low	150	42.4
	Medium	146	41.2
	High	58	16.4
Source of drinking water	Pipe water	4	0.02
	River water	141	39.8
	Thank water	209	59.0

**Abbreviation**: MUAC = mid upper arm circumference

### Prevalence of anemia

**Fig 1:**
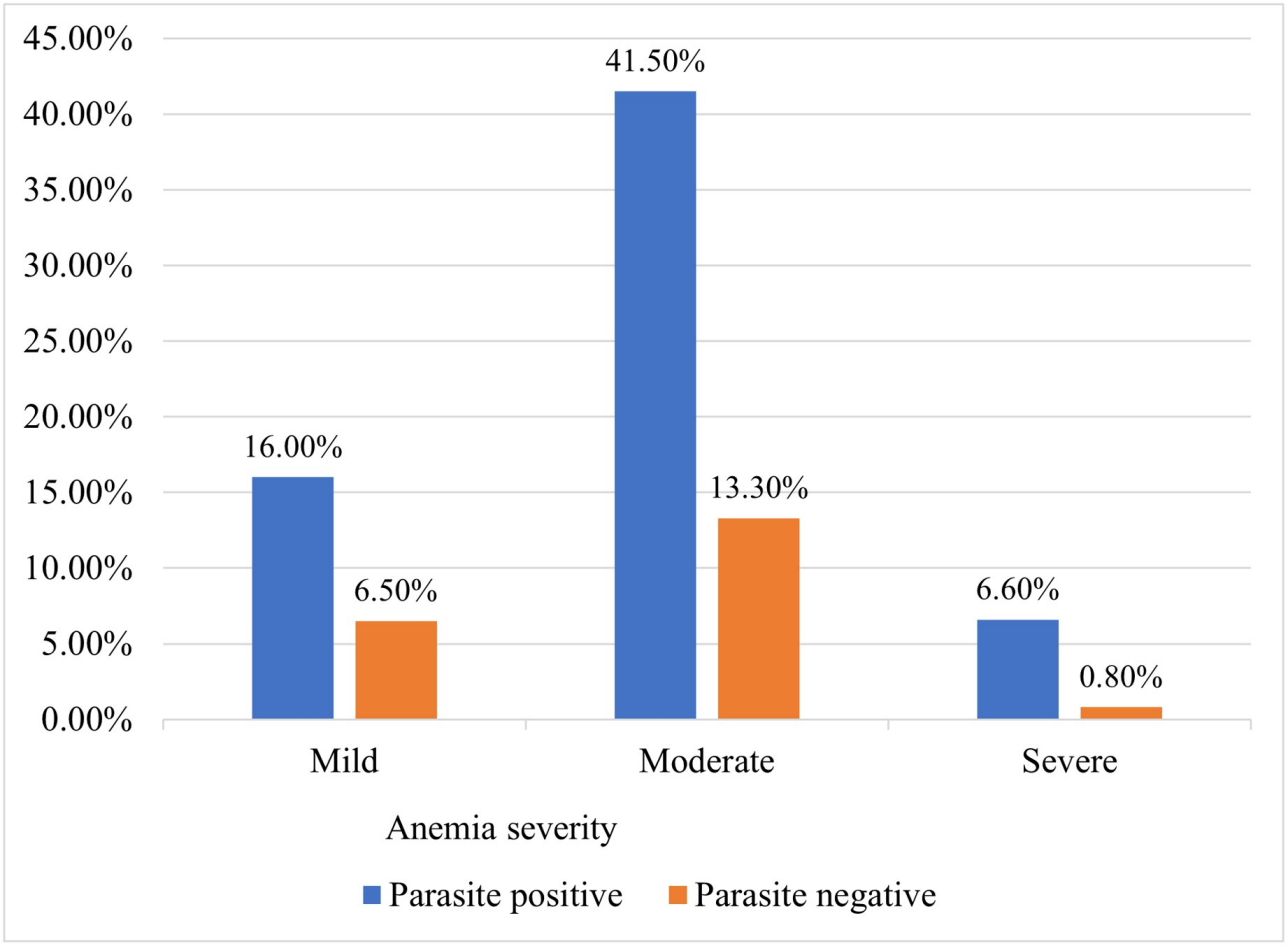
Anemia severity and parasitic infection among IDP children at debark refugee, 2022.

The overall prevalence of anemia among IDP children at debark refugee camp was 119 (33.62%; 95%CI: 28.7, 38.7). The prevalence of mild, moderate and severe anemia was 33 (9.3%), 77 (21.75%) and 9 (2.5%) respectively. Moderate anemia was predominant among children who infected with parasites 44 (41.5%) (Fig 1). The Hb level of the children ranged from 6.6 g/dl to 17.3 g/dL with a median (IQR) value of 11.9 (10.7–13.4) g/dL. The prevalence of anemia was found to be 98 (52.1%) among children who stayed in the camp more than 6 months. The magnitude of anemia was 45 (32.1%) and 33 (76.74%) among children who were stunted and wasted, respectively. Sixty-eight (64.15%) of children were anemic from those who infected with intestinal parasites (Table 3).

**Table 3:** The prevalence of anemia among IDP children at Debark refugee camp, 2022.

Variables	Category	Frequency (N)	Anemia N (%)	
			Yes	No
Sex	Male	194	129 (66.5)	65 (33.5)
	Female	160	106 (66.4)	54 (33.6)
Age of children	6 months-59 months	107	68 (63.5)	39 (36.5)
	5–11 years	187	123 (65.8)	64 (34.2)
	12–14 years	60	44 (73.3)	16 (26.7)
HAZ	Stunted	140	95 (67.9)	45 (32.1)
	Normal	214	10 (65.4)	74 (34.6)
MUAC	Wasted	43	10 (23.3)	33 (76.7)
	Normal	311	225 (72.4)	86 (27.6)
Maternal education	Un able to read and write	162	85 (52.5)	77 (47.5)
	Primary education	149	117 (78.5)	32 (21.5)
	Secondary and above*	43	33 (76.7)	10 (23.5)
Camp duration	< = 5 months	166	145 (87.4)	21 (12.7)
	> = 6 months	188	90 (47.9)	98 (52.1)
Presence of intestinal parasite	Yes	106	38 (35.9)	68 (64.2)
	No	248	197 (79.4)	51 (20.6)
Type of intestinal parasite	*A*.*lumbricoides*	46	22 (47.8)	24 (52.2)
	*G*.*lambilia*	42	12 (28.6)	30 (71.4)
	*E*.*histolotica*	11	4 (36.4)	7 (63.6)
	*Hookworm*	7	0	7 (100.0)
Presence of diarrhea	Yes	123	42 (34.4)	81 (65.6)
	No	231	193 (83.6)	38 (16.4)
Presence of fever	Yes	142	51 (35.9)	91 (64.1)
	No	212	184 (86.8)	28 (13.2)
Vomiting	Yes	26	6 (23.1)	20 (76.0)
	No	328	229 (69.8)	99 (30.2)
Meat consumption	Yes	70	63 (90.0)	7 (10)
	No	284	172 (60.6)	112 (39.4)
Egg consumption	Yes	79	69 (87.3)	10 (12.7)
	No	275	166 (60.4)	109 (39.6)
Fruit and vegetable consumption	Yes	28	24 (85.7)	4 (14.3)
	No	326	211 (64.7)	115 (35.3)
Milk intake	Yes	32	25 (78.1)	7 (21.9)
	No	322	210 (65.2)	112 (34.8)
Tea consumption	Yes	199	136 (68.3)	63 (31.7)
	No	155	99 (63.9)	56 (36.1)
Consumption of tea consumption (n = 119)	Before meal	44	33 (75.0)	11 (25.0)
	During meal	48	28 (58.3)	20 (41.7)
	After meal	107	75 (70.1)	32 (29.9)

**Abbreviations**: HAZ = height for age z-score, MUAC = mid upper arm circumference

Secondary and above* indicates including college and university

### Iron deficiency anemia

In this study, the prevalence of IDA among anemic children was 30/119 (25.2%; 95% CI: 18.1, 33.9). The median (IQR) value of SI and SF were 11.6 (7.2–21.8) μmol/L and 21.1 (9.2–36.9) μg/l, respectively. The prevalence of IDA among intestinal parasites infected children was 22 (32.4%) (Table 4).

**Table 4:** Prevalence of IDA from anemic IDP children at debark refugee camp (n = 119), 2022.

Variables	Category	Anemia Frequency	Prevalence of IDA N (%)	
			Non-IDA N (%)	IDA N (%)
Sex	Male	65	50 (76.9)	15 (23.1)
	Female	54	39 (72.2)	15 (27.8)
Age	6–59 months	39	33 (84.6)	6 (15.4)
	5–11 years	64	46 (71.2)	18 (28.1)
	12–14 years	16	10 (62.5)	6 (37.5)
Presence of intestinal parasite	Yes	68	46 (67.7)	22 (32.4)
	No	51	43 (84.3)	8 (15.7)
Presence of diarrhea	Yes	81	61 (75.3)	20 (24.7)
	No	38	28 (73.7)	10 (26.3)
Presence of fever	Yes	91	67 (73.6	24 (26.4)
	No	28	22 (78.6)	6 (21.4)
Consumption of fortified food	Yes	63	61 (96.8)	2 (3.2)
	No	56	28 (50.0)	28 (50.0)
Meat consumption	Yes	7	4 (57.1)	3 (42.9)
	No	112	85 (75.9)	27 (24.1)
Fruit and Vegetable consumption	Yes	4	4 (100.0)	0 (0.00)
	No	115	85 (73.9)	30 (26.1)
Egg consumption	Yes	10	5 (50.0)	5 (50.0)
	No	109	84 (77.1)	25 (22.9)
Tea consumption	Yes	63	47 (74.6)	16 (25.4)
	No	56	42 (75.0)	14 (25.0)
HAZ	Stented	74	31 (68.9)	14 (31.1)
	Normal	45	58 (78.4)	16 (21.6)
MUAC	Wasted	33	22 (66.7)	11 (33.1)
	Normal	86	67 (77.9)	19 (22.1)

**Abbreviations**: HAZ = weight for age z-score, IDA = iron deficiency anemia, MUAC = mid upper arm circumference

### Factors associated with anemia

Both bivariate and multivariate binary logistic regression were performed to determine the association between dependent and independent variable. Based on the bivariable binary logistic regression analysis, educational status of the care giver, shortage of food, children’s dietary diversity, meat consumption, fruit consumption, egg consumption, intestinal parasite infection, wasting, diarrhea, fever, selling of ration, and duration in the camp, were associated with the prevalence of anemia in bivariable analysis (Table 5). Variables with a p-value of <0.25 in bivariable analysis were selected for multivariable binary logistic regression. After multivariate logistic regression analysis, low dietary diversity (AOR = 4.9; 95%CI: 2.0, 11.7), staying in the camp six months and above (AOR = 4.2; 95% CI: 1.9, 9.4, having diarrhea in the last two weeks of the data collection period (AOR = 2.7; 95% CI: 1.3, 5.7), having fever in the last two weeks of the data collection period (AOR = 3.4; 95% CI: 1.6, 7.1), and being wasted (AOR = 3.6; 95% CI: 1.3, 10.3) remained significantly associated with anemia (Table 6).

**Table 5:** Bivariable logistic regression analysis of factor associated with anemia among IDP children at Debark refugee camp, 2022.

Variables	category	Anemia status		Crud odds ratio COR (95% CI)	P-value
		Anemic N (%)	Non anemic N (%)		
Age category	<5 years	39 (32.7)	68 (28.9)	1.00	
	5–11 years	64 (53.8)	123 (52.3)	0.91 (0.6, 1.5)	0.701
	12–14 years	16 (13.5)	44 (18.8)	0.6 (0.3, 1.27)	0.299
Sex of children	Male	65 (54.6)	129 (54.9)	1.00	
	Female	54 (45.4)	106 (45.1)	1.01 (0.6, 1.6)	0.961
HH size	< = 3	30 (5.2)	98 (41.7)	1.00	
	4–5	71 (59.6)	115 (48.9)	2.01 (0.8, 3.3)	0.661
	> = 6	18 (15.1)	22 (9.4)	2.67 (0.7, 5.6)	0.271
Number of children per HH	1	11 (9.2)	44 (18.7)	1.00	
	2	50 (42.0)	106 (45.1)	1.8 (0.89, 3.9)	0.293
	> = 3	58 (48.7)	85 (36.2)	2.7 (0.8, 5.7)	0.183
Camp duration	3–5 months	21 (17.6)	145 (61.7)	1.00	
	> = 6 months	98 (82.4)	90 (38.3)	7.5 (4.4, 12.8)	<0.001*
Educational Status of care giver	Unable to Read and write	77(47.5)	85 (52.5)	2.9 (1.4, 6.5)	0.005*
	Primary education	32 (21.5)	117 (78.5)	0.9 (0.40, 2.0)	0.800
	Secondary education *	10 (23.3)	33 (76.7)	1.00	
Marital status of care giver	Married	91 (76.5)	182 (78.4)	1.00	
	Single	28 (23.5)	50 (21.6)	1.1 (0.6, 1.9)	0.673
HAZ	Stented	45 (32.1)	95 (67.8)	1.1 (0.7, 1.7)	0.635
	Normal	74 (34.6)	140 (65.4)	1.00	
MUAC	Wasted	33 (76.7)	10 (23.2)	8.6(4.1, 18.3)	<0.001*
	Normal	86 (27.6)	225 (76.7)	1.00	
Presence of intestinal parasites	Present	68 (64.2)	38 (35.9)	6.9(4.18, 11.4)	<0.001*
	Absent	51 (20.5)	197 (79.4)	1.00	
Presence of fever	Yes	91(64.1)	28 (13.2)	11.7(6.9,19.8)	<0.001*
	No	51 (35.9)	184 (86.8)	1.00	
Presence of diarrhea	Yes	81(65.9)	42 (34.2)	9.7 (5.9,16.3)	<0.001*
	No	38 (16.5)	193(83.6)	1.00	
Meat consumption	Yes	7 (10.0)	63 (90.0)	1.00	
	No	112 (39.4)	172 (60.5)	5.8 (2.5, 13.3)	<0.001*
Fruit and vegetables consumption	Yes	4 (14.3)	24 (85.7)	1.00	
	No	115 (35.3)	211 (64.7)	3.2 (1.1, 9.6)	0.032*
Egg consumption	Yes	10 (12.6)	69 (87.3)	1.00	
	No	109 (39.6)	166 (60.4)	4.5 (2.2, 9.2)	<0.001*
DDVS	Low	100(66.7)	50 (33.3)	16.3(8.7, 30.2)	<0.001*
	Medium	16 (10.9)	130 (89.0)	1.00	
	High	3 (5.2)	55 (94.8)	0.4 (0.1, 1.5)	0.210*
Shortage of food	Yes	110 (43.4)	143 (56.5)	7.8 (3.8, 16.3)	<0.001*
	No	9 (8.9)	92 (91.1)	1.00	
Sell ration	Yes	61 (62.2)	37 (37.8)	5.63(3.4, 9.3)	<0.001*
	No	58 (22.6)	198 (77.3)	1.00	
Ration duration	< = 25days	96 (54.2)	81 (45.7)	7.9 (4.7, 13.4)	<0.001*
	26–30 days	23 (12.9)	154 (87.0)	1.00	

CI = confidence interval, COR = Crude odds ratio, DDVS = dietary diversity score, HAZ = height for age status, MUAC = mid upper arm circumference, Secondary education

* = includes college and above,

** = indicates statistical significance association in multivariable logistic regression.

**Table 6:** Factors associated with anemia in IDP children, at Debark refugee camp, 2022.

Variables	Categories	Anemia status		Bivariable analysis	Multivariable analysis	
		Anemic (%)	Nonanemic (%)	COR (95%CI)	AOR (95%CI)	p-value
Sex of child	Male	65 (33.5)	129 (66.5)	1.00		
	Female	54 (33.8)	106 (66.2)	1.0 (0.6, 1.6)		
Children’ age	6-59months	39 (36.5)	68 (63.5)	1.6 (0.7, 3.2)		
	5–11 years	64 (34.2)	123 (65.8)	1.4 (0.7, 2.7)		
	12–14 years	16 (26.7)	44 (73.3)	1.00		
Caregiver Education	Unable to Read and write	77 (47.5)	85 (52.5)	2.9 (1.4, 6.5)	0.78(0.2,2)	0.69
	Primary education	32 (21.5)	117 (78.5)	0.9 (0.40, 2.0)	0.7(0.2,2.4)	0.58
	Secondary education *	10 (23.3)	33 (76.7)	1.00	1.00	
Dietary diversity score	Low	100(66.7)	50 (33.3)	16.3(8.7, 30.2)	4.9(2.0,11.7)	<0.001**
	Medium	16 (10.9)	130 (89.0)	1.00	1.00	
	High	3 (5.2)	55 (94.8)	0.4 (0.1, 1.5)	0.5 (0.1, 2.7)	
Camp duration	> = 6 months	98 (52.1)	90 (47.9)	7.5 (4.4, 12.8)	4.2 (1.9, 9.4)	<0.001**
	3-5months	21 (12.7)	145 (87.4)	1.00	1.00	
Intestinal parasites	Present	68 (64.2)	38 (35.9)	6.9(4.18, 11.4)	2.1 (0.9, 4.5)	0.06
	Absent	51 (20.5)	197 (79.4)	1.00	1.00	
Diarrhea	Yes	81(65.9)	42 (34.2)	9.7 (5.9,16.3)	2.7(1.3,5.7)	0.008**
	No	38 (16.5)	193(83.6)	1.00	1.00	
Fever	Yes	91(64.1)	28 (13.2)	11.7(6.9,19.8)	3.4 (1.6,7.1)	0.001**
	No	51 (35.9)	184 (86.8)	1.00	1.00	
Selling ration	Yes	61 (62.2)	37 (37.8)	5.63(3.4, 9.3)	1.5 (0.7, 3.2)	0.30
	No	58 (22.6)	198 (77.3)	1.00	1.00	
Ration duration	< = 25days	96 (54.2)	81 (45.7)	7.9(4.7, 13.4)	1.9 (0.8, 4.3)	0.12
	26–30 days	23 (12.9)	154 (87.0)	1.00	1.00	
Food shortage	Yes	110 (43.4)	143 (56.5)	7.8 (3.8, 16.3)	0.57(0.17,1.9)	0.365
	No	9 (8.9)	92 (91.1)	1.00	1.00	
HAZ	Stented	45 (32.1)	95 (67.8)	1.1 (0.7, 1.7)		
	Normal	74 (34.6)	140 (65.4)	1.00		
MUAC	Wasted	33 (76.7)	10 (23.2)	8.6(4.1, 18.3)	3.6 (1.3,10.3)	0.016**
	Normal	86 (27.6)	225 (76.7)	1.00	1.00	
Fruit consumption	Yes	4 (14.3)	24 (85.7)	1.00	1.00	
	No	115 (35.3)	211 (64.7)	3.2 (1.1, 9.6)	0.6 (0.2, 2.8)	0.600
Meat consumption	Yes	7 (10.0)	63 (90.0)	1.00	1.00	
	No	112 (39.4)	172 (60.5)	5.8 (2.5, 13.3)	1.1 (0.3, 3.4)	0.993
Egg consumption	Yes	10 (12.6)	69 (87.3)	1.00	1.00	
	No	109 (39.6)	166 (60.4)	4.5 (2.2, 9.2)	1.1 (0.4, 3.3)	0.812

AOR = Adjusted odds ratio, CI = confidence interval, COR = Crude odds ratio, MUAC = mid upper arm circumference, Secondary education

* = includes college and above,

** = indicates statistical significance association in multivariable logistic regression

## Discussion

Anemia prevalence data continue to be an important indicator of public health problems since it is related to morbidity and mortality, particularly in more vulnerable and nutritionally deficient groups such as displaced children [30]. The overall prevalence of anemia among IDP children at Debark refugee camp was 33.62% (95%CI: 28.7, 38.7), this value is a moderate public health problem. As compared to the global prevalence of anemia among children the current study suggests lower prevalent than WHO report, which is 39.9% (10).

In this study, the prevalence of anemia was comparable with studies conducted in Bhutaness refugee camp which was (34.3%) [31], Gaza strip, Palestine (35.3%) [32], Beirut, Lebanon (30.5%) [33], Klang Valley, Malaysia, refugee camp (37.8%) [34]. The comparable and moderate prevalence of anemia in these setting might be due to that displacement increases the risk of nutritional deficiency, overcrowding, infectious diseases and these phenomena, which may aggravate the onset of anemia in comparable way even though some sort of difference in terms of geographic, ethnic and socio-economic characteristics. However, the prevalence of anemia in this study is lower than the study conducted in Kebribeyah refugee camp, Somalia region which was (52.4%) [11]. The possible reason for the low prevalence of anemia in this study compared to the previous study may be the duration in the camp. Children who enrolled in this study had less than one year camp duration, while in the above study they stayed more than a year. According to our finding, it was indicated that IDP children who stayed more than six months were develop more anemia than children stayed shorter period. Long-term reliance on limited food diversity may increase the prevalence of anemia in the previous study. The other possible explanation for the discrepancies in magnitude of anemia may be deference in study population. The previous study that mentioned above were only include under five children, while this study includes under fifteen children and in fact young children are more susceptible for anemia due to the increased requirement of iron for growth and development, so this may increase the prevalence of anemia in the aforementioned studies when compared to our study.

The current finding was also lower than studies conducted in African IDPs and refugee camp such as South Sudan refugee camp (40.0%) [35], Edo State, Nigeria (54.0%) [12], Cameroon (84.0%) [13], and Asian countries, like Kutupalong Camp, Bangladesh (47.9%) [36], Burma (72.6%) [37], Jablia refugee (59.7%) [38], Za’atri camp in Jordan (48.4%.) [39]. The deference in anemia prevalence may be attributed to the high infection burden in the previous study and geographical difference. Malaria has been showed a major cause of anemia in endemic areas by causing hemolysis of infected and uninfected erythrocytes and bone marrow dyserythropoiesis [40]. The study area is not malaria endemic, thus there were only two cases of malaria recorded, and this may have contributed to the reduced prevalence of anemia in our study. For example, study conducted in Nigeria IDP children prevalence of malaria was reported to be 55.2%, so this may contributed to the high prevalence of anemia in that study (54.0%) [12].

The prevalence of anemia seen in this study was higher than studies conducted at refugee camps of newly arriving government-assisted refugee children in Canada (15.7%) [41], United Nations Relief and Works Agency for Palestine Refugees in the Near East (UNRWA) (25.0%) [42], Dekalb country, Georgia refugee (17.7%) [43], and Greece, (13.7%) [44]. The possible reason for discrepancies in anemia prevalence between the present study and the previous studies may be related to ethnic, geographic variability as well as deference in socio- economic status and availability of health insurance in the refugee population in which study were conducted. Most of the aforementioned refugee camps are found in high income countries and the general health facilities and accessibility of nutritional demand may be better. Most of these refugees are reception camps from different countries of the world with diverse ethnic group that might be the reason in the difference between our study and the previous studies.

In this study, moderate anemia was higher than the other types of anemia (21.6%). Similarly with this finding the previous studies conducted in Kebribeyah refugee camp Somali region, Ethiopia (48.1%) [11], Edo state, Nigeria (36%) [12] and Cameroon (52.4%) [13] also report high prevalence of moderate anemia than other types of anemia. The prevalence of severe anemia in this study was 2.5% which is lower than the study conducted in refugee camp of Myanmar 10.9% [45], Burma refugee camp (10.4) [46], One reason for difference in severe anemia prevalence would be, the cut off value for Hb to determine anemia severity. Some study mentioned above categorized severe anemia when the Hb level below 8mg/dl for under five children, however, in this study the cut of value for severe anemia in under five children was 7mg/dl. So, this may increase the prevalence rate of severe anemia in the previous study. The other major possible explanation may be high infection burden in other study like malaria may increase the severity of anemia.

In the current study, the prevalence of IDA from anemic children was reported to be 25.2%. Iron deficiency anemia is in comparable with the previous study conducted in Gaza strip, Palestine (33.5%) [38]. On the other hand, this finding was much lower than the study conducted in Burma refugee (64.9%) [46]. The low prevalence of IDA in our study compared to the aforementioned study is due to shorter camp duration. Children in the previous study stayed with the minimum of one year, which is longer period than our study participants camp duration, and long reliance on the camp food stocks may increase the risk of IDA. In many refugee settings, the usual diet contains low iron, but high levels of iron absorption inhibitors, such as phytates, found in rice, and long period consumption of these foods may contribute the high prevalence of IDA by inhibiting iron absorption [47].

In the current study dietary diversity score was found significantly associated with anemia among IDP children of debark refugee camp. Children with low dietary diversity score were 4.9 times (95%; CI: 2.0, 11.7) more likely to be anemic when compared to children with the medium dietary diversity score. One strategy for raising children’s consumption of micronutrients is to encourage a variety of foods. Consuming a variety of meals from various food categories provides vital nutrients [48], and phytochemicals for appropriate growth and development as well as the prevention of diseases [49]. However, refugee camp children have limited access to nutrition and low dietary diversity results in malnutrition [50]. The introduction of foods with low iron levels during foods transition and elevated frequencies of infectious and parasitic diseases among refugee children, are also important factors in determining childhood anemia [50].

Children who stayed in the camp six months and above were 4.2 times (95%; CI: 1.90, 9.4) more likely to be anemic as compared to IDP children with 3–5 months camp duration. The higher prevalence in long stayed camp children might be due to food aid dependency. The most stapled food in this study area were rice and wheat, and it is believed to be rice contains high levels of iron absorption inhibitors, such as phytates [47]. As a result of long-time consumption of the same foods items in the camp may induce the onset of anemia. The other possible explanation for the high odds of anemia due to long duration of camp would be the gradual depletion of iron due to negative balance between daily iron need and in adequate micronutrient intake to balance the body need of iron for RBC synthesis [51]. Children stayed in the camp for longer period are vulnerable to communicable diseases such as parasitic infections due to overcrowding condition, poor sanitation and inaccessibility of pure water. Parasitic infection utilizes most micronutrients including iron and block its absorption to the body. The unavailability of those essential nutrient due to parasitic infection increases the risk of having anemia through camp duration [52].

Children with current history of diarrhea or last two weeks of data collection were 2.7 times (95%; CI: 1.3, 5.7) more likely to had anemia than those without diarrhea. The findings of this study was supported by previous studies conducted in Palestinian refugee camps [32], and Kebribeyah refugee camp, Somalia region [11]. Children who experience diarrheal attacks have worsened physical and nutritional conditions. These children are prone to malnutrition through nutrient loss, due to this reason those children are vulnerable to anemia. The other possible mechanism is, during subsequent diarrhea condition, iron and other mineral deficiency can develop, because diarrhea affects the small intestine’s ability to absorb nutrients from their meals. So, those Children can have high tendency to nutritional anemia [53].

In this finding children who had fever in last 2 weeks of the study were 3.4 times (95%; CI: 1.6, 7.1) more likely to develop anemia. Even though only two cases of malaria were reported in our study, there might be other infectious diseases that may increases the presence of fever. Fever is one of the usual clinical features that appear during the course of several infectious diseases such as, parasitic, bacterial protozoal infections [54]. In refugee children this infection burden is high [55]. Fever can develop by the body for adaptive immune response against these infections and infestations [54]. Due to infection hepcidin level will increase result in inhibiting iron absorption from the enterocyte and may result in anemia.

In the current study, wasting was found significantly associated with anemia. Children whose MUAC was less than the WHO cut of value (wasted) were 3.6 times (95%; CI: 1.3, 10.3) more likely to be anemic when compared with non-wasted children. The high prevalence of wasting in younger children (16.82%) may increase onset of anemia in this study. Several mechanisms can elucidate the higher odds of anemia among malnourished children. First, as an adaptive reaction, malnourished children have lower metabolism. Reduced metabolism results in less need for oxygen and reduced production of red blood cells, which can lead to the development of adaptive anemia [56]. The second possible reason might be, wasted children are more likely to be deficient in micronutrients including iron, which is the most important micronutrient for RBC synthesis. In developing countries, particularly in refugee camp children, diverse supply of foods is limited and they are at risk of developing anemia [57]. The other possible mechanism related to anemia may be wasted children have lowered immunity, which makes them more susceptible to infectious diseases. The infection then causes loss of micronutrients, absorption blockage, underutilization of bioavailable nutrients such as iron, blood loss and also immune-mediated destruction of RBCs would be associated with the onset of anemia [56].

The study was limited in determination of red cell indices like MCV, RDW, MCHC, and HCT for the classification of anemia. Secondly, concentration technique for intestinal parasite examination was not performed. Despite these limitations, it is the first cross-sectional study, which tried to show the prevalence and associated factors of anemia and IDA by assessing SF and SI, among conflict hit area in Ethiopia, which gives better emphasis to know nutritional status particularly iron through displaced and asylum-seeking children.

## Conclusions

In this study, anemia was found to be moderate public health problem among IDP children at debark refugee camp. Moderate anemia is more prevalent cases in this study. There is also relatively high prevalence of IDA was reported in the current stud. The increased prevalence of anemia in IDP children is due to different factors. In this study, low dietary diversity score, long duration in the camp, diarrhea, fever, and wasting were significantly associated with anemia. Generally, diet or nutritional deficiency particularly acute malnutrition, communicable diseases due to overcrowding were high in Debark refugee children and these contributes this moderate prevalence of anemia in our study setting. Therefore, during emergency condition like conflict, the Ethiopian government should formulate a policy towards to IDP children to reduce anemia and iron deficiency anemia by preventing contributing factors like wasted, long stayed camp children, low dietary intake, symptomatic like fever and diarrheal children. Food aid organizations and refugee camp managers need consider iron rich foods and heme iron sources and vegetables to enhance iron rich foods intake by the households. Refugee camp children by the help of their care mother/giver should be visit, complain and get early treatment from camp health center, while they fell any illness such as diarrhea, vomiting, fever. A health professional should also consider providing health education and consultation for the households. They also should focus on preventing of communicable diseases in the camp.

## Supporting information

S1 File English version of questionary with information sheet. (DOCX)

S2 File Amharic version of questionary with information sheet. (DOCX)

S3 File Anemia data of IDP children. (DTA)

## Abbreviations

BF: Blood Film
CRP: C- Reactive Protein
DDS: Dietary Diversity Score
FAO: Food Agriculture Organizations
HAZ: Height for Age
Hb: Hemoglobin
HHs: Households
ID: Iron Deficiency
IDA: Iron Deficiency Anemia
IDMC: Internal Displacement Monitoring Center
IDP: Internal Displacement Person
IQR: Inter Quartile Range
MUAC: Mid Upper Arm Circumference
NGO: Non-Governmental Organizations
RBCs: Red Blood Cells
SF: Serum Ferritin
SI: Serum Iron
SOP: Standard Operating Procedures
UNHCR: United Nation High Commissioner for Refugees
WHO: World Health Organization
