# Supplementary material for: Anemia and associated factors among internally displaced children at Debark refugee camp, North Gondar, Northwest Ethiopia
Source: PLoS One. 2023 May 10;18(5):e0285627. doi: 10.1371/journal.pone.0285627 (PMC10171677; doi:10.1371/journal.pone.0285627)
Supplement: S1 File — (DOCX) [file pone.0285627.s001.docx]

**Information sheet**

**Informed Consent form (English)**

Greeting:

My name is ______________________ (data collector’s name), I am here to collect information from you about child related issue and taking a blood sample (near to 3ml) to determine the level of your Hgb and anemia status.

If you grant us consent, we will do a laboratory examination for you which will be done by medical personnel. This will help us to assess the hemoglobin status of child. Blood collection from you will be used for the further analysis. If there will any abnormal finding you will be informed and contact to get health care facility.

**Personal agreement statement**

I the undersigned bellow, have understood the above information, which has been fully explained to me by the data collector. I agree to take part in this study.

Participant’s signature ________________ Date _______________

Data collector signature Date

**Informed Assent Form**

I have read the information above, or it has been read to me. I have been given the opportunity to ask questions and my questions have been answered to my satisfaction. I voluntarily consent that my child participates in this study provided he/she gives assent. I agree with the researcher to collect her/his blood and be a participant in this study. And I understand that I have the right to withdraw my child from the study at any time

Guardians’ name

Relationship to participant Signature Date:

Data collector name: Signature: Date:

Name of investigator _____________Signature ___________Date of investigation __________

**Questionnaire (English version**)

**Part I: Socio demographic information of children**

| Code | Questions | Response | Skip to |
| --- | --- | --- | --- |
| Q01 | Age of your child | __________ years |  |
| Q02 | Sex of your child | 1. Male  2. Female |  |
| Q03 | How many are there in your household? | ______________ |  |
| Q04 | Number of children per household | ______________ |  |
| Q05 | How long have you been in this camp? In years or months | ______ |  |
| **Part II: Socio demographic characteristics of family/care giver** | | | |
| Q06 | Age | ___ years |  |
| Q07 | Sex | 1. Male  2. Female |  |
| Q08 | Marital status | 1. Married 2. Separated 3. Widowed 4. Divorced |  |
| Q09 | Educational status | 1. No formal education 2. Primary school 3. Secondary school 4. College/ University |  |
| Q10 | Relationship to child | 1. Mother 2. Female care giver 3. Father 4. Male care giver |  |

**Part III -Children’s food habit**

| **Code** | | **Questions** | | **Response** | | **Skip to** |
| --- | --- | --- | --- | --- | --- | --- |
| Q11 | Have you ever had food shortage? | | | 1. Yes  2. No | |  |
| Q12 | How many times do you eat per day? | | | 1. More than 3 times 2. Three times 3. Two times 4. One times | |  |
| Q13 | Have you eaten meat and meat product? | | | 1. Yes  2. No | |  |
| Q14 | How frequent do you eat meat and meat products | | | 1. Once a week 2. Twice a week 3. More than twice a week 4. Once per month 5. Less than one times per month | |  |
| Q15 | Did you drink tea? | | | 1. Yes 2. No | | If ‘No’ skip to Q23 |
| Q16 | When did you drink Tea? | | | 1. Before meal 2. After meal 3. During meal | |  |
| Q17 | How frequently do you use tea? | | | 1. More than 2 times a day 2. Once per day 3. Once per week 4. More than once per week 5. Less than once per week | |  |
| Q18 | How frequently do you use fruits and vegetable like Orange, Papaya, Mango and Banana, green vegetable? | | | 1. Once a week 2. Twice a week 3. More than twice per week 4. Once per month 5. Less than one time per month 6. Not at all | |  |
| Q19 | How frequently do you use egg? | | | 1. Every day 2. Once a week 3. Twice a week 4. Once per month 5. Less than one time per month 6. Not at all | |  |
| Q20 | How frequently do you use Milk and milk products | | | 1. More than once a day 2. Once a day 3. Once per week 4. Less than per week 5. Not at all | |  |
| Q21 | What is the staple diet in the family? | | | 1. Rice and pasta 2. Maize and sorghum 3. Wheat 4. Enjera 5. Others specify ______ | |  |
| Q22 | Have you ever received ration in your family? | | | 1. Yes  2. No | |  |
| Q23 | If yes, for how many days did you use the general ration? | | | _____________ | |  |
| Q24 | Did you use all the general ration? | | | 1. yes  2. No | |  |
| Q25 | Have you sold or share the general ration | | | 1. yes  2. No | |  |
| Q26 | Did your child consumed fortified food during the last 24 hors? | | | 1. yes  2. No | |  |
| **Part IV: Health and physiologic condition of the child** | | | | | | |
| Q27 | Did you have any repeated diarrhea for these 2 weeks? | | | 1. Yes 2. No | |  |
| Q28 | Did you have any repeated vomiting in the last two weeks | | | 1.yes  2. No | |  |
| Q29 | Did you have any repeated fever for these 2 weeks? | | | 1. Yes 2. No | |  |
| Q30 | What is your main source of water? | | | 1. Piped water 2. Well water  3. River water 4. Rain water  5. Tank water 6. Other specify | |  |
| **Part V: Pallor assessment** | | | | | | |
|  | Site of pallor | | Sex | Age | Mark “X” if there is pallor | |
| 01 | Conjunctivae | |  |  |  | |
| 02 | Eye | |  |  |  | |
| 03 | Skin | |  |  |  | |
| 03 | Palm | |  |  |  | |

**Part VII: Food dietary diversity questionary**

| Code | Food group | Examples | YES=1  NO=0 | source:   1. purchase 2. gift 3. other (specify) |
| --- | --- | --- | --- | --- |
| 01 | Cereals | Millet, sorghum, maize, rice, or other grains (beside general ration) |  |  |
| 02 | Vegetables | Pumpkin, carrots, sweet potatoes, green pepper, tomato |  |  |
| 03 | Fruits | Orange, papaya, mango banana |  |  |
| 04 | Organ meat (iron-rich) | liver, kidney, heart or other organ meats or blood -based foods |  |  |
| 05 | flesh meats | Lamb, goat, chicken … |  |  |
| 06 | Eggs |  |  |  |
| 07 | fish and sea foods |  |  |  |
| 08 | legumes, nuts and seeds | beans, peas, lentils, nuts, seeds or foods made from these |  |  |
| 09 | milk and milk products | milk, cheese, yogurt or other milk products |  |  |
| 10 | oils and fats | oil, fats or butter added to food or used for cooking |  |  |
| 11 | Sweets | sugar, honey, sweetened soda or sugary foods such as chocolates, sweets or candies |  | ` |
| 12 | Spices, Condiments, Beverages | sauces, coffee, tea, beverage |  | ` |

**Individual dietary diversity score calculation form**

| Starchy Samples | DGLV | Other VitA f/v | Other f/v | Organ meat | Flesh meat | Eggs | Legumes/ Nuts | Milk | Total  DDVS |
| --- | --- | --- | --- | --- | --- | --- | --- | --- | --- |
|  |  |  |  |  |  |  |  |  |  |
