# Supplementary material for: Anemia and associated factors among internally displaced children at Debark refugee camp, North Gondar, Northwest Ethiopia
Source: PLoS One. 2023 May 10;18(5):e0285627. doi: 10.1371/journal.pone.0285627 (PMC10171677; doi:10.1371/journal.pone.0285627)
Supplement: S2 File — (DOCX) [file pone.0285627.s002.docx]

**የአዋቂዎች የስምምነት መጠየቂያ ቅጽ**

ስለ ጥናቱ መረጃ በመረጃ ሰብሳቢው ተብራርቶልኛል፡፡ የዚህ ጥናት ዓላማም በሰሜን ምዕራብ ኢትዮጵያ በደባርቅ የተፈናቃዮች ካምፕ ውስጥ በሚኖሩ ህጻናት ላይ ያለውን የደም ማነስ ስርጭትን እና ተያያዥ ምክንያቶችን መገምገም መሆኑን ተረድቻለሁ፡፡ የምሰጠው የደም ናሙናም በጤናየ ላይ ምንም አይነት ጉዳት እንደሌለው ተረድቻለሁ፡፡ በማንኛውም ጊዜ በመካከል እና በጥናቱ ለመሳተፍ ፈቃደኛ ካልሆንኩ ተሳትፎየን የማቆም መብት እንዳለኝም ተብራርቶልኛል። እኔ ከታች በፊርማየ ያረጋገጥኩት ከላይ ያለው መረጃ በመጃ ሰብሳቢው ሙሉበሙሉ ተብራርቶልኛል፡፡ ስለሆነም በዚህ ጥናት ለመሳተፍና ተገቢውን መረጃ ለመስጠት ተስማምቻለሁ፡፡

የተሳታፊ ፊርማ ________________ ቀን _______________

የመረጃ ሰብሳቢ ስም ፊርማ ቀን

**የልጆች አሳዳጊዎች/ተንከባካቢዎች የስምምነት መጠየቂያ ቅጽ**

እኔ የልጆቹ ወላጅ/ አሳዲጊ ስሆን፡በስደተኞች ካምፕ ውስጥ በልጆች ላይ ያለውን የደም ማነስ ስርጭት እና የተያያዙ ምክንያቶች ምርመራ ጥናት ላይ ልጄ የጥናቱ ተሳታፊ እንድትሆን/እንዲሆን በሙሉ ፈቃዴ ወስጃለሁ፡፡ ከዚህ ጋር የተያያዘውን የመግለጫ ቅጽ በትክክል አንብቤ/ተነቦልኝ ተረዴቻለሁ፡፡ ልጄ ላይ ስለሚደረግ ማንኛውም ጥናት ተገንዝቤአለሁ፡፡ በተጨማሪም አስፈላጊውን ገለጻና ማብራሪያ ከላይ በተጠቀሱት ሰው ተደርጎልኛል፡፡ አጥኚዎቹ ለደም ማነስ ምርመራ ከሚወሰደዉ ደም በታጨማሪ የደም ናሙና ከልጄ ጣት ወይም ክንድ ላይ እንደሚወስደ በሚገባ ተረድቻለዉ፡፡ ነገርግን የደም ናሙና በሚወሰድበት ወቅት ሊፈጠር የሚችለውን አነስተኛ ህመምና የደም መፍሰስ ለማስወገድ ልምድ ባላቸው እና ስልጠና በተሰጣቸው ባለሙያዎች እንደሚከናወን ተረድቻለሁ፡፡ በማንኛውም ጊዜ ከጥናቱ ላይ ልጄን ማግለል እንደምችል አውቄያለሁ፡፡ ከልጄ የሚሰበሰበው ማንኛውም መረጃ በጥንቃቄና ሚስጥራዊነቱ በተጠበቀ ቦታ እንደሚቀመጥ አውቄያለሁ፡፡ ስለዚህም ልጄን በጥናቱ ውስጥ ለማሳተፍ በፍፁም ፈቃድኝነት የስምምነት ቃሌን መስጠቴን በፊርማዬ አረጋግጣለሁ፡፡

የተንከባካቢው/ዋ ፊርማ ቀን

የመረጃ ሰብሳቢ ስም ፊርማ ቀን

**ክፍል አንድ ስለ ማህበረሰብ አቀፍ እና-ስነ-ሕዝብ የልጆች አጠቃላይ መረጃ**

ውድ ምላሽ ሰጪዎች፣ በዚሁ መሰረት ትክክለኛ መረጃ እንድትሰጡ በአክብሮት እንጠይቃለን።

ስለ ጊዜዎ እና ተሳትፎዎ እናመሰግናለን።

የተሳታፊዎች ኮድ ቁጥር _________________

**ስለ ማህበረሰብ አቀፍ እና-ስነ-ሕዝብ የልጆች አጠቃላይ መረጃ**

| መለያ ቁጥር | ጥያቄዎች | መልስ | ይዝለሉ |
| --- | --- | --- | --- |
| 01 | የልጅዎ ዕድሜ ስንት ነው | __________ ዓመት |  |
| 02 | ጾታ | 1. ወንድe 2. ሴት |  |
| 03 | የቤተሰብ ብዛት | ______________ |  |
| 04 | በቤተሰብ ውስጥ የልጆች ብዛት ስንት ነው; | ______ |  |
| 05 | በዚህ ካምፕ ውስጥ ምን ያህል ጊዜ ቆዩ? በአመታት ወይም በወር ውስጥ | ______ |  |
| **ክፍል ሁለት** ስለ ማህበረሰብ አቀፍ እና-ስነ-ሕዝብ የናቶች ወይም የተንከባካቢ አጠቃላይ መረጃ | | | |
| 06 | እድሜ | ___ አመት |  |
| 07 | ጾታ | 1. ወንድ 2. ሴት |  |
| 08 | የጋብቻ ሁኔታ | 1. ያገባ/ች 2. ያላገባ/ች 3. የተፋታ/ች  4. ሚስት የሞተችበት/ባል የሞተባት |  |
| 09 | የትምህርት ደረጃ | 1. መደበኛ ትምህርት የለም 2. የመጀመሪያ ደረጃ ትምህርት ቤት 3. ሁለተኛ ደረጃ ትምህርት ቤት 4. ኮሌጅ / ዩኒቨርሲቲ |  |
| 10 | ከልጅዎ ጋር ያልዎት ዝምድና | 1.እናት  2. ሴት ተንከባካቢ  3. አባት  4. ወንድ ተንከባካቢ |  |

**ክፍል አራት** የልጆች አመጋገብ ልማድ

| **ኮድ** | | **ጥያቄ** | **መልስ** | **ዝለል** |
| --- | --- | --- | --- | --- |
| 11 | ልጅዎ የምግብ እጥረት አጋጥሞ/ሟት ያውቃል; | | 1. አዎ 2. አያውቅም |  |
| 12 | ልጅዎ በቀን ስንት ጊዜ ይመገባል/ለች? | | 1. ከ 3 ጊዜ በላይ 2. ሶስት ጊዜ  3. ሁለት ጊዜ 4. አንድ ጊዜ |  |
| 13 | ልጅዎ ስጋና የስጋ ምርቶችን ደጋግሞ ይመገባል/ትመገባለች | | 1. አዎ 2. የለም |  |
| 14 | ስጋ እና የስጋ ምርቶችን ምን ያህል ደጋግመው ይበላሉ | | 1. በሳምንት አንድ ጊዜ 2. በሳምንት ሁለት ጊዜ  3. በሳምንት ከሁለት ጊዜ በላይ 4. በወር አንድ ጊዜ  5. በወር ከአንድ ጊዜ ያነሰ 6. በጭራሽ አይመገቡም |  |
| 15 | ልጅዎ ሻይ ይጠጣል/ለች? | | 1. አዎ 2. አይ |  |
| 16 | ሻይ መቼ ነዉ የሚጠጡት? | | 1. ከምግብ በፊት 2. ከምግብ በኋላ 3. ከምግብ ጋር |  |
| 17 | ሻይ ምን ያህል ጊዜ ይጠቀማሉ? | | 1. በቀን ከ 2 ጊዜ በላይ 2. በቀን አንድ ጊዜ  3. በሳምንት አንድ ጊዜ 4. በሳምንት ከአንድ ጊዜ በላይ 5. በሳምንት ከአንድ ጊዜ ያነሰ |  |
| 18 | እንደ ብርቱካን፣ ፓፓያ፣ ማንጎ እና ሙዝ ያሉ ፍራፍሬዎችን ምን ያህል ጊዜ ይጠቀማሉ? | | 1. በሳምንት አንድ ጊዜ 2. በሳምንት ሁለት ጊዜ  3. በሳምንት ከሁለት ጊዜ በላይ 4. በወር አንድ ጊዜ 5. በወር ከአንድ ጊዜ ያነሰ 6. በጭራሽ አልጠቀምም |  |
| 19 | ምን ያህል በተደጋጋሚ እንቁላል ይጠቀማሉ | | 1. በየቀኑ 2. በሳምንት አንድ ጊዜ  3. በሳምንት ሁለት ጊዜ 4. በወር አንድ ጊዜ  5. በወር ከአንድ ጊዜ ያነሰ ጊዜ  6. በጭራሽ አልጠቀምም |  |
| 20 | ምን ያህል ጊዜ ወተት እና የወተት ተዋጽኦዎችን ይጠቀማሉ | | 1. በቀን ከአንድ ጊዜ በላይ 2. በቀን አንድ ጊዜ  3. በሳምንት አንድ ጊዜ 4. ከሳምንት ያነሰ  5. በጭራሽ አይጠቀሙም |  |
| 21 | በቤተሰብ ውስጥ ዋናው የምግብ ምንጭ ምንድነው? | | 1. ሩዝ እና ፓስታ 2. በቆሎ እና ማሽላ  3. ስንዴ 4. እንጀራ  5. ሌሎች ደግሞ ______ን ይገልጻሉ። |  |
| 22 | የእርስዎ ቤተሰብ ባለፉት የምግብ ራሽን ተቀብሏል? | | 1. አዎ 2. አልተቀበልንም |  |
| 23 | አዎ ከሆነ፣ ያገኙት አጠቃላይ ራሽን ለምን ያህል ጊዜ ቆየ? | | ______ |  |
| 24 | ሁሉም ራሽን በእርስዎ ቤተሰብ ይጠቀሙታል? | | 1. አዎ 2. አንጠቀምም |  |
| 25 | የተሰጡትን ራሽን ይሽጣሉ? | | 1. አዎ 2. አንሸጥም |  |
| 26 | ልጅዎ ባለፉት 24 ሰዓት ውስጥ የተቀነባበሩ ምግቦችን እንደ ፕላምፕኔት እና የመሳሰሉትን ተጠቅመው ያውቃል/ታውቃለች? | | 1. አዎ 2. የለም |  |

| **ክፍል አምሥት** የልጆችየ ጤና እና የፊዚዮሎጂ ሁኔታዎች | | | | | |
| --- | --- | --- | --- | --- | --- |
| **ኮድ** | **ጥያቄዎች** | | | **መልስ** | **ይዝለሉ** |
| 27 | ልጅዎ ለእነዚህ 2 ሳምንታት ተደጋጋሚ ተቅማጥ ነበረበት/ነበረባት? | | | 1. አዎ 2. የለም |  |
| 28 | ባለፉት ሁለት ሳምንታት ውስጥ ልጅዎ ተደጋጋሚ ማስታወክ ነበረበት/ነበረባት? | | | 1. አዎ 2. የለም |  |
| 29 | ባለፉት ሁለት ሳምንታት ውስጥ ልጅዎ ትኩሳት ነበረው/ነበራት? | | | 1. አዎ 2. የለም |  |
| 30 | የመጠጥ ውሃ ከየት ነው የምትጠቀሙት? ከአንድ በላይ ካለ አክብብ | | | 1. የቧንቧ ውሃ 2. የጉድጓድ ውሃ 3. የወንዝ ዉሃ 4. የዝናብ ውሃ  5. የታንከር ውሃ 6. ሌላ ይግለጹ ______ |  |
| **የፓለር ምርመራዎች** | | | | | |
| ፓሎር የታየበት ቦታ | | እድሜ | ጾታ | ዉጤት ‘’X’’ በማድረግ ይግለጹ |  |
| 01 | አይን ልባስ ላይ |  |  |  |  |
| 02 | ጥፍር ላይ |  |  |  |  |
| 03 | መዳፍ ላይ |  |  |  |  |
| 04 | ፊት ላይ |  |  |  |  |
| 04 | ቆዳ ላይ |  |  |  |  |

የቃለ ምልልሳችን መጨረሻ ይህ ነው ፣ ስለ ትብብርዎ እናመሰግናለን!

**ክፍል ስድስት** የልጆች የምግብ እና አመጋገብ ስብጥር ጥያቄዎች

| ባለፉት 24 ሰአት ዉስጥ ከሚከተሉት አንዱን የምግብ አይነት ከተጠቀሙ 1 ን ይምረጡ ከልተጠቀሙ ደግሞ 0 ን ይምረጡ | | | | |
| --- | --- | --- | --- | --- |
| ተ.ቁ | የምግብ አይነቶች | ለምሳሌ ያክል | እጠቀማለሁ = 1 አልጠቀምም = 0 | ምንጭ፡-  1. ግዢ 2. ስጦታ 3. በብድር  4. እርዳታ |
| 01 | Cereals | ማሽላ፣ በቆሎ፣ ሩዝ ወይም ሌሎች እህሎች (ከአጠቃላይ ራሽን በተጨማሪ) |  |  |
| 02 | ጥራጥሬዎች | ለውዝ፣ ባቄላ፣ አተር፣ ምስር የመሳሰሉትን |  |  |
| 03 | በቫይታምን የበለጸጉ አትክልቶች | ዱባ፣ ካሮት. ስኳር-ድንች፣ ቲማቲም |  |  |
| 04 | በቫይታሚን የበለጸጉ ፍራፍሬዎች | ብርቱካን፣ ፓፓያ፣ ማንጎ እና ሙዝ |  |  |
| 05 | አረንገዴ ቅጠላማ አትክልቶች | ቃሪያ፣ጎመን እና የመሳሰሉትን |  |  |
| 06 | በብረት ንጥረ ነገር የበለጸጉ የስጋ አይነቶች | ጉበት፣ ኩላሊት፣ ልብ ወይም ሌሎች |  |  |
| 07 | ሌሎች ጠቦት የስጋ አይነቶች | የበግ ስጋ ፣ የፍየል ስጋ ፣ የዶሮ ስጋ |  |  |
| 08 | እንቁላል |  |  |  |
| 09 | ዓሳ |  |  |  |
| 10 | ወተትና የወተት ተዎጽኦዎች | ወተት፣ አይብ፣ እርጎ ወይም ሌሎች የወተት ተዋጽኦዎች |  |  |
| 11 | ዘይቶችና ቅባቶች | ዘይት፣ ስብ፣ ወይም ቅቤ ወደ ምግብ የተጨመረ ወይም ለማብሰያ ይጠቅማሉ |  | ` |
| 12 | ጣፋጭ ነገሮች አና ቅመሞችና መጠጦች | ስኳር፣ ማር፣ ወይም እንደ ቸኮሌት፣ ጣፋጮች ወይም ከረሜላ የመሳሰሉ ጣፋጭ ምግቦች፤ ስጎ፣ቡና፣ ሻይ |  | ` |
